# Investigation of blood flow in the external carotid artery and its branches with a new 0D peripheral model

**DOI:** 10.1186/s12938-016-0133-x

**Published:** 2016-02-04

**Authors:** Yoshihito Ohhara, Marie Oshima, Toshinori Iwai, Hiroaki Kitajima, Yasuharu Yajima, Kenji Mitsudo, Absy Krdy, Iwai Tohnai

**Affiliations:** Department of Oral and Maxillofacial Surgery, Yokohama City University Graduate School of Medicine, 3-9 Fukuura, Kanazawa-ku, Yokohama, 236-0004 Japan; Department of Interfaculty Initiative in Information Studies, The University of Tokyo, 7-3-1 Hongo, Bunkyo-ku, Tokyo, 113-0033 Japan; Institute of Industrial Science, The University of Tokyo, 4-6-1 Komaba, Meguro-ku, Tokyo, 153-8505 Japan

**Keywords:** Blood flow, External carotid artery and its branches, 0D peripheral model, Computational fluid dynamics, Simulation, Oral cancer, Intra-arterial chemotherapy

## Abstract

**Background:**

Patient-specific modelling in clinical studies requires a realistic simulation to be performed within a reasonable computational time. The aim of this study was to develop simple but realistic outflow boundary conditions for patient-specific blood flow simulation which can be used to clarify the distribution of the anticancer agent in intra-arterial chemotherapy for oral cancer.

**Methods:**

In this study, the boundary conditions are expressed as a zero dimension (0D) resistance model of the peripheral vessel network based on the fractal characteristics of branching arteries combined with knowledge of the circulatory system and the energy minimization principle. This resistance model was applied to four patient-specific blood flow simulations at the region where the common carotid artery bifurcates into the internal and external carotid arteries.

**Results:**

Results of these simulations with the proposed boundary conditions were compared with the results of ultrasound measurements for the same patients. The pressure was found to be within the physiological range. The difference in velocity in the superficial temporal artery results in an error of 5.21 ± 0.78 % between the numerical results and the measurement data.

**Conclusions:**

The proposed outflow boundary conditions, therefore, constitute a simple resistance-based model and can be used for performing accurate simulations with commercial fluid dynamics software.

## Background

The standard treatment for oral cancer is surgery, but radical surgery for advanced oral cancer often causes severe oral dysfunction, including speech and swallowing disorders. However, organ and function preservation can be maximized by adopting a multidisciplinary approach that combines radiotherapy and chemotherapy [1]. Selective or superselective intra-arterial chemotherapy, in particular, plays an important role in obviating the need for radical surgery. However, the side effects can be severe and the distribution of the anticancer agent to tumour-feeding arteries is unclear. Therefore, it is necessary to clarify the optimal dose of anticancer agent for each patient, and it is critical to obtain accurate information on flow distribution in each vessel for optimal drug delivery. Even though the final target of this study is to understand the distribution of the anticancer agent in the carotid artery area, including the branches of the external carotid artery (ECA) which act as feeding arteries for oral cancer, the present study focuses on patient-specific blood flow simulations in the area relevant for intra-arterial chemotherapy since anticancer agent flows are correlated with the blood flow due to their small concentration. Despite the recent progress of measurement methods, such as ultrasound-based methods, it is still difficult to obtain accurate information about the flow distribution in vessels, such as branches of the ECA, in deep soft tissues.

Due to remarkable progress over the last two decades, computational methods have allowed us to conduct realistic haemodynamic simulations. Simulations have also recently been performed in the fields of neurosurgery and cardiovascular surgery for atherosclerosis and aneurysm [2–18]. Simulation is also used as a preoperative examination tool [19–23]. More recent patient-specific 3D model studies have been oriented to the carotid haemodynamics in order to establish correlation between blood flow-induced wall shear stress and atherosclerosis using patient-specific carotid models [24–29]. Using patient-specific internal carotid artery (ICA) model, mechanical stresses also has been analysed by blood flow simulation [30]. In this regard, simple and versatile computational haemodynamic simulation using patient-specific imaging data before treatment is expected to improve treatment quality and allow surgeons to predict treatment results. Also, by using patient-specific imaging data, the postoperative outcome can be compared with the preoperative state. However, it is difficult for surgeons to conduct numerical simulations since they require a certain set of skills related to computers and numerical algorithms, which are outside the scope of clinical practice. Therefore, the use of simulation as a tool to assist treatment can be very challenging. Furthermore, the simulation must yield accurate results within a reasonable time in order for it to be applicable to individual patients.

To achieve a more realistic simulation, the boundary conditions should include the effects of the entire circulatory system. Although a number of numerical models for these boundary conditions have been developed, most models, such as the Windkessel model [31–33] and the lumped parameter model [34–36], rely on physiological parameters, such as resistance, compliance and conductance, which can be determined only from measurement data. Another approach is to conduct a multi-scale simulation combined with a one dimensional–zero dimensional (1D–0D) simulation including the entire circulatory system developed by Liang et al. [37]. Although the multi-scale simulation with the entire circulatory system is more physiologically accurate, it is complicated and time consuming. Since our region of interest is limited to the head and neck area, it is not necessary to conduct a simulation of the entire circulatory system. However, it is necessary to consider the influence of the peripheral blood vessels. Therefore, in this study, we develop a systematic method to determine physiological parameters, such as the resistance of the peripheral network, based on anatomical and physiological knowledge and model the boundary conditions with a 0D model, in order to simulate the effects of the entire circulatory system. The 0D resistance model of vessels is created in such a way that the peripheral vessel network is constructed as a binary symmetric tree that is attached to the outlets of a three-dimensional (3D) geometric structure. As a result, the resistance model defines a relationship between pressure and flow at the outlets such as the outflow boundary conditions in the 3D blood flow simulation. Since the peripheral vessel network of the ECA consists of smaller blood vessels, they tend to be mechanically stiff so the model takes into account changes in the ratio of vessel length to diameter, obtained either from medical imaging data or from anatomical knowledge of smaller vessels. The proposed model also considers non-Newtonian effects in blood viscosity with respect to changes in vessel diameter by using the constitutive relation between the apparent viscosity of blood and the haematocrit.

Because it is more convenient to use commercial software rather than custom-developed software in clinical studies, we have developed a simple boundary condition which can be implemented in commercial software. The model was applied to two patients in order to investigate the flow rate in branching arteries over a large number of cardiac cycles. A comparison between the numerical simulations and the ultrasound measurement data of the flow rates in the superficial temporal artery (STA) of the two patients was performed.

## Methods

This study was reviewed and approved by the Ethics Committee of Yokohama City University (No. B110512003). Written informed consent to use image data was obtained from oral cancer patients.

### Patient-specific geometric model

#### Computed tomography angiography

Patients with oral cancer underwent computed tomography (CT) angiography before intra-arterial chemotherapy. We used a 64-slice CT scanner (Aquilion 64; Toshiba Medical Systems, Tokyo, Japan), and non-ionic contrast medium (100 ml) was injected at a rate of 4.0 ml/s through an antecubital vein with an automatic power injector. A bolus-tracking technique was used to select the individual scan delay of the arterial phase. Repetitive low-dose scans were performed with a delay of 8 s at a level inferior to the carotid bifurcation. To measure the bolus arrival time, the region of interest was chosen to lie in the common carotid artery (CCA). As soon as an enhancement level of 90 Hounsfield units was reached, the scanning procedure started automatically. For the arterial phase scan, the scanning volume included the inferior margin of the thyroid cartilage/bottom of C6 and the superior margin of the orbit. The scanner settings were 120 kV, 250 mA, 64 × 0.5 mm slice collimation, table speed 20.5 mm/rotation (pitch 0.641), and rotation time 0.75 s. The resolution matrix was 512 × 512 pixels with a slice thickness of 1 mm.

#### Patient-specific model

**Fig. 1:**
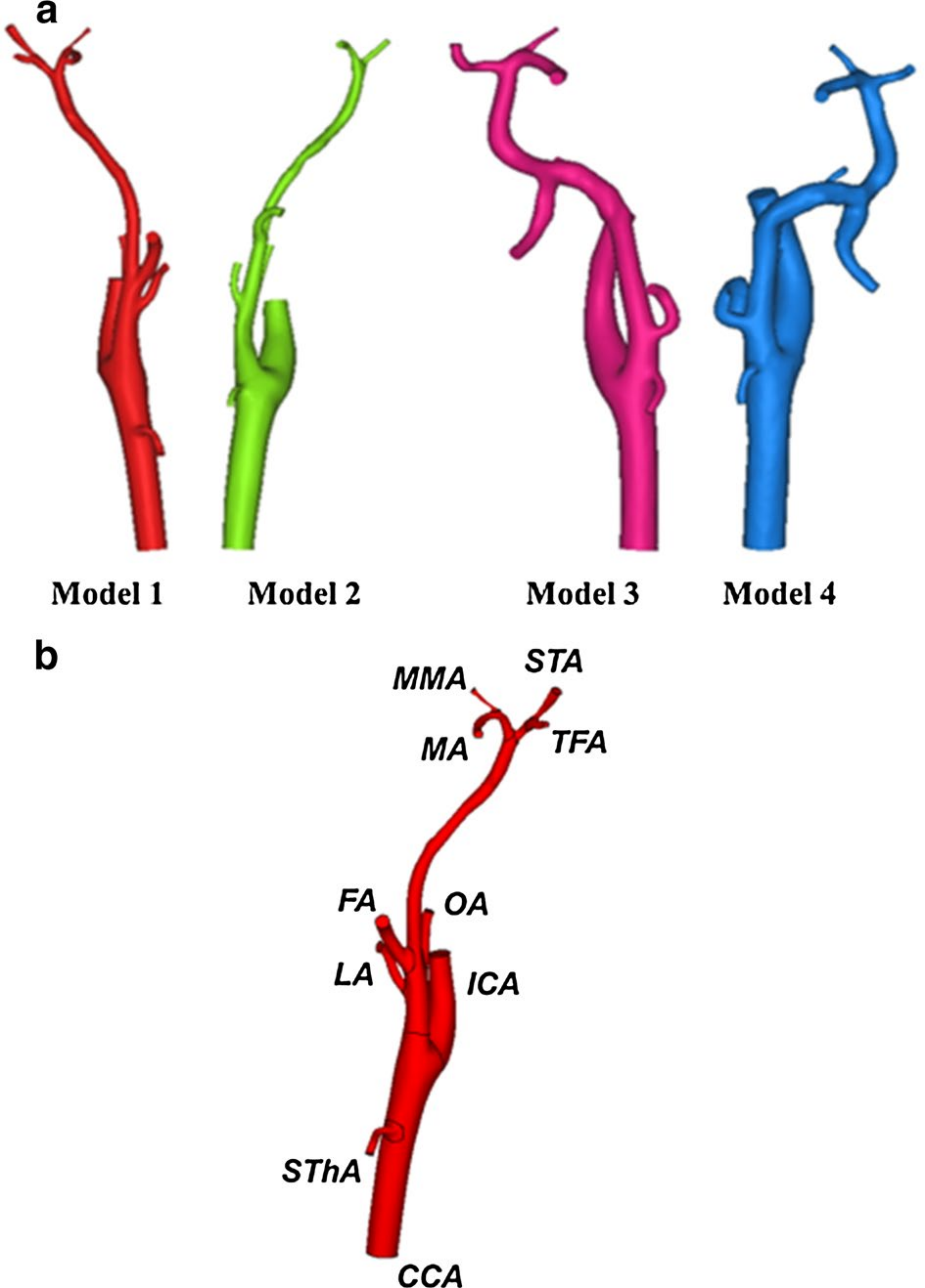
3D models constructed from computed tomographic angiography images (**a**) and branches of the external carotid artery in the analysis area (**b**). The figure shows the common carotid artery (CCA), the internal carotid artery (ICA), the superior thyroid artery (SThA), the lingual artery (LA), the facial artery (FA), the occipital artery (OA), the maxillary artery (MA), the superficial temporal artery (STA), the middle meningeal artery (MMA) and the transverse facial artery (TFA)

Digital imaging and communication in medicine (DICOM) data from the CT angiography of the two patients (right lower gingival cancer and right tongue cancer) were input into Mimics software (Materialise, Leuven, Belgium). The bilateral carotid arteries of each patient were segmented, and thus four separate three-dimensional patient-specific analysis models were created (Fig. 1a, b). The models included the CCA, the ICA, the ECA and ECA branches, such as the superior thyroid artery (SThA), lingual artery (LA), facial artery (FA), occipital artery (OA), maxillary artery (MA), posterior auricular artery (PAA), STA, middle meningeal artery (MMA) and transverse facial artery (TFA). Due to limitations in CT resolution, models 1 and 2 included no PAA, models 2–4 had no TFA, and model 2 had no MMA. Therefore, models 1, 3 and 4 had nine branches, and model 2 had seven branches.

#### Mesh generation

**Fig. 2:**
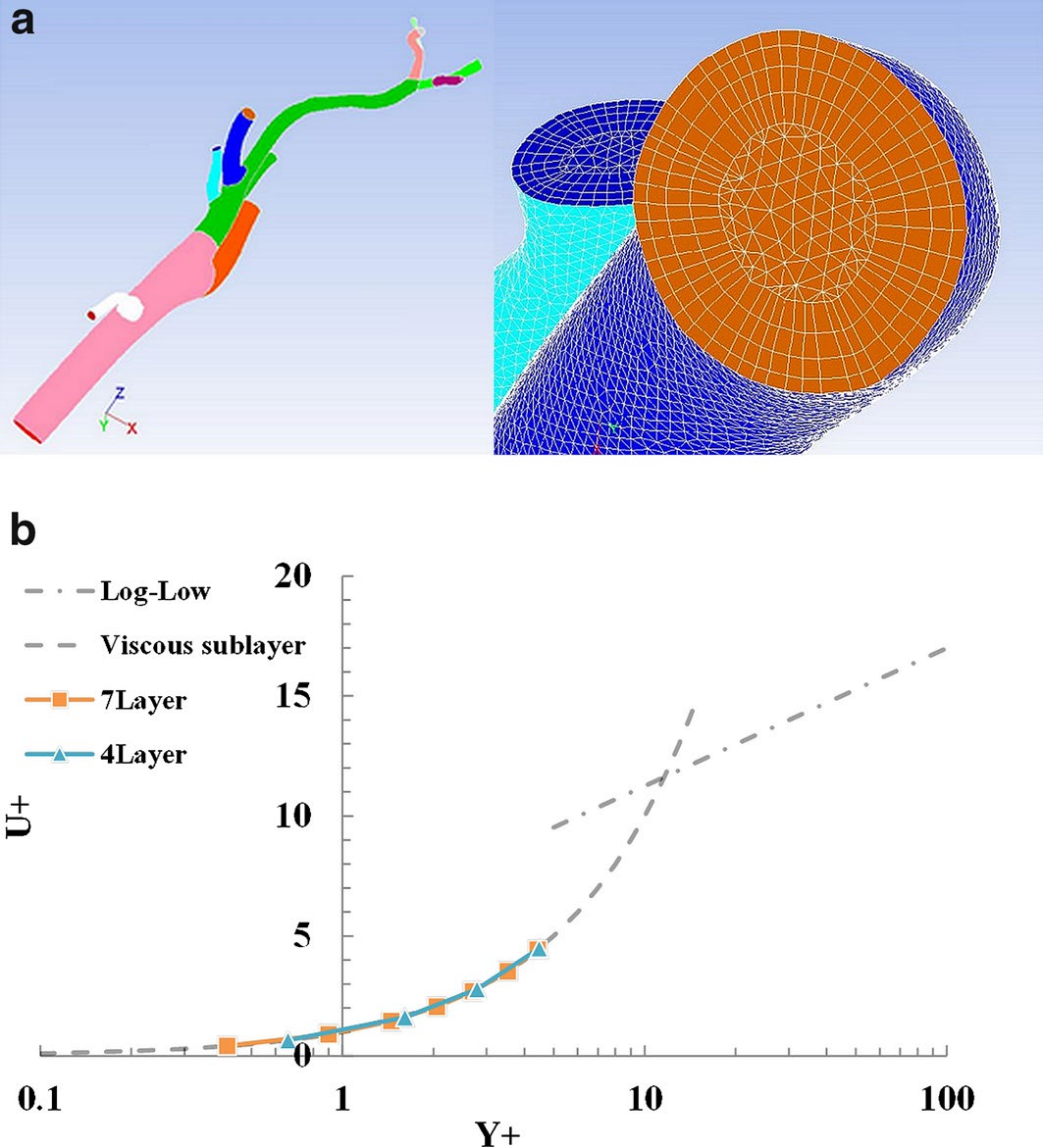
Tetrahedral mesh with a prismatic boundary layer (**a**) and mesh value of y+ less than one in each vessel (**b**)

Before mesh generation for a simulation, the diameters of each artery immediately after branching were measured. In addition, each artery was cut off at a length 5 times the diameter in order to minimize the influences of both inflow and outflow boundary conditions on the flow distributions (Fig. 2a). Numerical analyses with the finite volume method (FVM) were performed using the general-purpose fluid dynamics software FLUENT (Ansys Inc, Canonsburg, PA). The meshes for the FVM were generated using ICEM-CFD software (Ansys Inc., Canonsburg, PA). The mesh consists of tetrahedral cells in the artery core and prismatic cells in the region near the artery wall, as shown in Fig. 2a, and the total number of cells was approximately 3,000,000. Due to this mesh configuration, the cells in the core region are regular while orthogonality was maintained near the wall. In order to resolve the boundary layer, it is necessary to create a fine mesh near the wall, but the calculation time results in increase. Because we suppose to use as a future clinical tool, the mesh requires to minimize within a range that does not affect the present analysis. Therefore, we adopted a tetra prism type mesh. In this paper, the first grid point from the wall was located within y^+^<1, as shown in Fig. 2b. There were four layers of prismatic cells in the viscous layer, and the mesh resolution was good enough for the analysis.

### Outflow boundary conditions

We performed 3D blood flow simulations using three types of outflow boundary conditions as the alternative boundary conditions for patient-specific vessels models to be described later in detail: (1) zero-pressure outflow boundary conditions, (2) the pressure boundary conditions with the conventional 0D resistance model and (3) the pressure boundary conditions with the present 0D resistance model. In both 0D resistance models, a capillary with a radius of 12 μm or less was set to be the end of a branch. The conventional model used a constant *λ* (the ratio of the vessel length to its radius of 30) while *λ* in the present model varied depending on a size of a blood vessel diameter. Also, the terminal resistance in the present model was adjusted so that the terminal pressure became 30 mmHg, which is within the physiological range of the blood pressure. In two types of 0D resistance model, the flow rates acquired from the 3D analysis became the inflow boundary condtions for the 0D analysis while the pressure from the 0D analysis became the outflow boundary conditons for the 3D analysis. For the zero-pressure boundary condition, the pressures in all outlets were fixed at zero pressure in all outlets.

### Numerical methods

Since the 3D simulation was conducted by regarding blood as an incompressible Newtonian fluid (1050 kg/m^3^ density and 0.0046 Pa•s viscosity) in laminar flow with the average properties of blood, blood vessel walls can be regarded as rigid and the no-slip condition can be applied. Inflow boundary conditions of a constant blood flow velocity (Ultrasound system) were prescribed. Since the time scale of administration of drugs in intra-arterial chemotherapy is at least 1 h, a steady state blood simulation is performed instead of an unsteady pulsatile simulation. Three types of outflow boundary conditions for pressure on the outlets (0D resistance models) were prescribed.

The finite volume commercial software Ansys FLUENT was used to carry out steady states fluid dynamics simulations. A SIMPLE scheme was used to couple velocity and pressure and the discretization was accurate to the second order. The 0D resistance models are used as input data for user-defined functions in the code.

Simulations were performed on a PC running the Microsoft Windows 7 Professional operating system. The CPU was a dual-core Intel Xeon W5590 (clock frequency: 3.33 GHz) and there was 64 GB of RAM per core. The convergence of the simulations was verified by monitoring the mass flow rate of each outlet and the three velocity components. Convergence was achieved when each of these monitored values and the residuals showed oscillations of the order of 10^−4^ or less around a constant mean value. Most of the simulations required a total wall-clock time of less than 26 h.

### Mathematical modelling of outflow boundary conditions

#### Physiological background

**Fig. 3:**
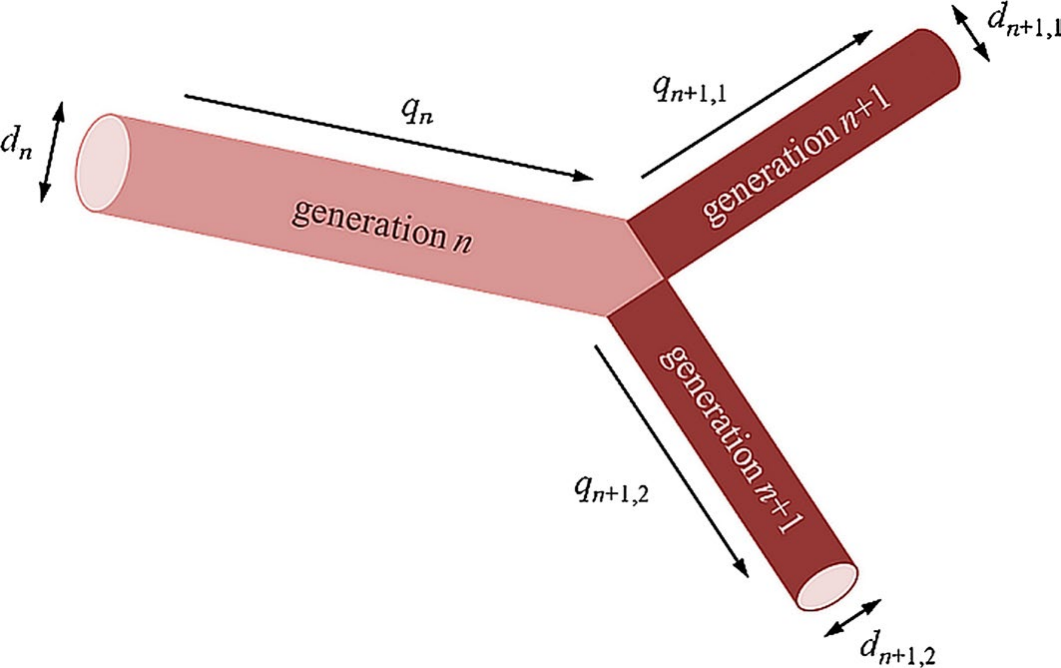
A parent vessel splitting into two daughter vessels

**Fig. 4:**
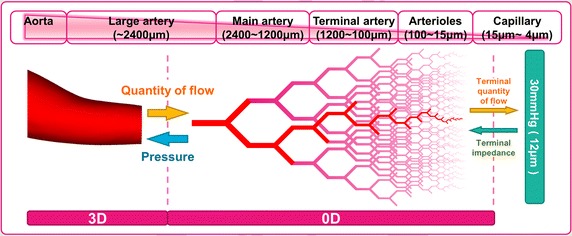
Anatomical components in the 0D resistance model

In order to achieve a realistic blood flow simulation, it is important to model appropriate physiological conditions, particularly boundary conditions. Even though the systemic arteries are compliant, vessels become stiffer with a smaller radius. Blood vessels are organized in a bifurcating tree in which the total cross-sectional area of the vessels expands from approximately 5 cm^2^ at the aortic root to approximately 400 cm^2^ at the arterioles [38]. This expansion in the total cross-sectional area occurs despite the decrease in diameter of individual vessels because the number of daughter vessels doubles at each bifurcation, as shown in Fig. 3. As the number of bifurcations increases, the flow resistance becomes higher and the pressure falls as blood flows through the arterial tree. In this paper, we adopt outflow boundary conditions based on a 0D resistance model to reflect the effects of the peripheral network on the 3D blood flow simulation; this allows us to conduct simulations using commercial software within a reasonable computation time. Since the diameter of the outlet in the 3D model varies, our 0D resistance model is divided into six groups (aorta, large arteries, main artery branches, terminal artery branches, arterioles and capillaries) depending on vessel diameter, as summarized in Fig. 4.

#### Geometrical modelling of the peripheral vessel network

The resolution of CT or magnetic resonance (MR) imaging is not sufficiently high for accurate geometric modelling of a peripheral blood vessel network with small arteries, arterioles and capillaries. Instead, the peripheral network is constructed with a self-similar geometry. The parent blood vessel of generation *i* has a diameter *d*_*i*_, and the flow rate *q*_*i*_ is divided into two branches in two daughter generations (*i* + *1, 1*) and (*i* + *1, 2*), as shown in Fig. 3. The geometry of peripheral blood vessels network of the 0D model is built on the basis of fractal due to anatomical knowledge [39]. In this study, we assume that the flow rate is distributed equally between the two symmetrical daughter vessels [40]:

1 $$q_{i} = q_{i + 1,1} + q_{i + 1,2}$$

2 $$q_{i + 1,1} = q_{i + 1,2} = 0.5q_{i},\quad i = 1, \ldots, n$$

where *n* denotes the total number of generations.

The diameters of the daughter generations are determined in accordance with a power law. This relation was suggested by Uylings [41] and was derived on the basis of the principle of minimum energy in the arterial system:

3 $$d_{i}^{\varepsilon } = d_{i + 1,1}^{\varepsilon } + d_{i + 1,2}^{\varepsilon }$$

4 $$d_{i + 1,1}^{\varepsilon } = d_{i + 1,2}^{\varepsilon } = 0.5d_{i}^{\varepsilon }$$

where ε = 3.0 for laminar flow and ε = 2.33 for turbulent flow. In this study, ε was set to 3.0 because the Reynolds number was smaller than 1000.

#### 0D resistance model

The outflow boundary conditions were developed as a 0D model to represent only the resistance of smaller arteries in the peripheral vessel network because this is easy to implement in the software used for the flow simulation. Since the peripheral network consists of relatively small blood vessels, the influence of resistance can be assumed to be more pronounced than that of compliance [42]. However, the elasticity of the arterioles and capillaries was reduced to reflect their properties.

Since the length of small arteries cannot be determined by medical imaging, many authors have discussed methods for determining the length of vessels. For example, Olufsen et al. [43] considered the ratio of vessel length to vessel radius *λ* = *l/r* and set the ratio *λ* to 50 ± 10 [44–46]. In the 0D models developed by Olufsen et al. [43], it was possible to produce a physiological pressure drop, but it was not possible to calculate the physiological pressure. Therefore, in this research, to calculate the physiological pressure, a pressure of 30 mmHg was applied to the capillary vessel domain at the end of the conventional 0D models. In the basic experiment, we calculated the applied physiological pressure to the peripheral vessel with *λ* fixed at 20, 30, 40 or 50. As a result, the average outlet pressure in 3D became 75, 88, 100, or 122 mmHg. The average physiological pressure from arteries to arterioles would be too high at 100 mmHg, and excessively low at 75 mmHg. Therefore, in the conventional 0D resistance model used for comparison in this research, the value of *λ* was set to 30, and terminal resistance values were set to 0 at the end of the network of blood vessels.

We can write the relationship between pressure drop and flow rate in the time domain as follows:

5 $$\frac{{P_{n} \left( L \right)}}{{Q_{n} \left( L \right)}} = R_{n} \left( L \right)$$

where *P*, *Q* and *R* denote pressure, quantity of flow and resistance, respectively, and the suffix *n* denotes the generation of bifurcation. The present resistance model involves two steps, as described above.

The first step is to calculate the resistance *R*_*i*_ (0) at the inlet of the artery at the *i*-th generation:

6 $$R_{i} \left( 0 \right) = \frac{8\,\mu \,\lambda }{{\left( {\pi r_{i}^{3} } \right)}} + R_{i} \left( L \right)$$

where *μ* is the viscosity of blood, *λ* is the ratio of the vessel length to its radius, and *R*_*i*_(*L)* is the resistance at outlet at the *i*-th generation, in which *L* is the distance from the inlet to the outlet at the *i*-th generation.

At a bifurcation point in the peripheral network, the resistance follows the same logic as resistance in electric circuits. If we assume that the parent vessel bifurcates into two daughter vessels symmetrically, the relation between the resistances of the parent and daughter vessels is given by

7 $$\frac{1}{{R_{i} \left( L \right)}} = \frac{1}{{R_{i + 1,1} \left( 0 \right)}} + \frac{1}{{R_{i + 1,2} \left( 0 \right)}}$$

The second step is to compute the resistance *R*_*i*_ (*L*) at the outlet of the artery at the *i*-th generation:

8 $$R_{i} \left( L \right) = \frac{1}{2}R_{i + 1,1} \left( 0 \right)$$

We categorized the vessels in the peripheral network into six groups based on their diameter and length, in accordance with a previous study [47], and the average value of *λ* for each group was determined. If the radius of the artery at the *i*-th generation indicates that the vessel is in the *k*-th group, *λ*_*i*_ is given by the value of *λ* for the *k*-th group shown in Table 1.

**Table 1:** Diameter, length and ratio of vessel length to vessel radius (*λ*) of each vessel in our peripheral network

Blood vessel	Ave. diameter (mm)	Min. diameter (mm)	Length (mm)	*λ*
ICA (all mean)	5.4834	4	117.3925	50.0154
Large arteries	6.5	2	200	61.5385
Main artery branches	2.4	1.2	100	83.3333
Terminal artery branches	1.2	0.1	10	16.6667
Arterioles	0.1	0.015	2	40
Capillaries	0.008	0.004	1	250

The values for the diameter and length of the internal carotid artery (ICA) are mean values over the four 3D models, and the λ value of each vessel is calculated from its length and half its averaged diameter

The advantage of the 0D resistance model is its flexibility in terms of setting the terminal resistance to a value in the physiological range, which is impossible with the structure impedance model [43, 48, 49]. The terminal resistance is simply given by

9 $$R_{n} \left( L \right)_{terminal} = \frac{{p_{terminal} }}{{Q_{n} \left( L \right)_{terminal} }}$$

Equation (9) allows us to update the terminal resistance at every step to match the physiological conditions of *P*_*terminal*_. The terminal pressure is set in accordance with medical data [49, 50]. These modifications to the 0D resistance model allow for the tree model to be extended to the terminal radius (12 μm in our model), where the pressure was set to 30 mmHg (Fig. 4).

The special shape of the ICA, which does not branch until it enters the skull, was not included in this study as it lies outside of the region of interest. Therefore, in this study, the length and diameter of the ICA were determined on the basis of actual medical data.

### Modelling of non-Newtonian viscosity

In large and medium arteries, blood behaves as a Newtonian fluid, and the viscosity. Blood has usually been treated as a Newtonian fluid in CFD studies of large and medium arteries. It does not exhibit a constant viscosity at all flow rates and is especially non-Newtonian in the microcirculatory system. Blood also exhibits non-Newtonian behaviour in small branches and capillaries, where the cells squeeze through and a cell-free skimming layer reduces the effective viscosity through the tube. To take into account non-Newtonian effects, the apparent viscosity is given as a function of both the diameter and the haematocrit [47, 51–53]. First, the haematocrit is given by

10 $$\begin{aligned} Hct = \left\{ {\begin{array}{*{20}c} {0.45} \\ {0.45\left( {0.196\,\,\log d - 0.117} \right)} \\ \end{array} } \right.\;\;\begin{array}{*{20}c} {\text{if}} \\ {\text{if}} \\ \end{array} \begin{array}{*{20}c} {d > 300\rm{\mu m}} \\ {d \le 300\rm{\mu m}} \\ \end{array} \end{aligned}$$

where *d* is the vessel diameter.

When the vessel diameter becomes smaller than 10 μm, the apparent viscosity decreases in accordance with the Fåhraeus–Lindqvist effect. By the inverse Fåhraeus–Lindqvist effect, the apparent viscosity increases when the vessel diameter is larger than a certain value. Equation (10) incorporates both effects.

**Fig. 5:**
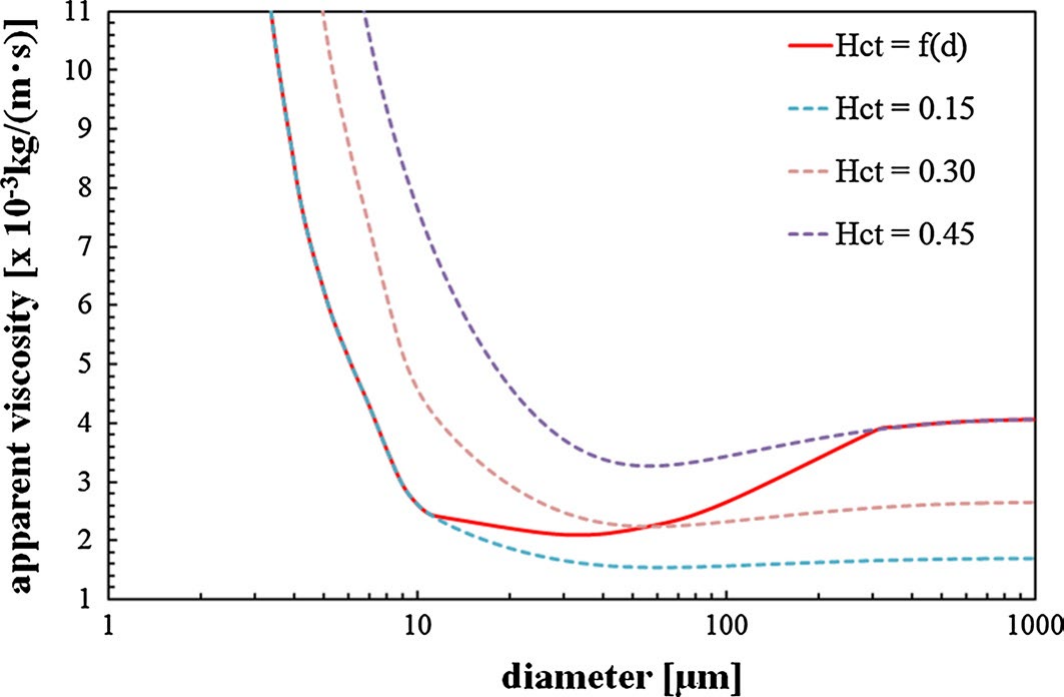
Apparent viscosity with variable haematocrit

The red line (*Hct* = *f* (*d*)) in Fig. 5 shows the behaviour of *Hct* in this study. The apparent viscosity is given in previous studies [54, 55]. According to Freitas [47], the haematocrit changes when the diameter *d* becomes smaller than 300 μm. We used Eq. (11), which was proposed by Pries et al. [54, 55], to obtain the following relation for the changes in the viscosity *μ*_*app*_ associated with changes in vessel diameter *d*.

11 $$\mu_{app} = \left[ {1 + \left( {\mu_{0.45} { - }1} \right)\frac{{\left( {1 - Hct} \right)^{Cd} }}{{\left( {1 - 0.45} \right)^{Cd} }}\left( {\frac{d}{{d{ \,-\, }1.1}}} \right)^{2} } \right]\left( {\frac{d}{{d{ \,-\, }1.1}}} \right)^{2}$$

where

12 $$Cd = \left( {0.8 + \exp \left[ { - 0.075\,d} \right]} \right)\left( {{ - }1 + \frac{1}{{1 + 10^{ - 11} d^{12} }}} \right) + \frac{1}{{1 + 10^{ - 11} d^{12} }}$$

and

13 $$\mu_{0.45} = 6\exp \left[ {{ - }0.085\,d} \right] + \mu_{Newtonian} { - }2.44\exp \left[ {{ - }0.06\,d^{0.645} } \right]$$

When *μ* (the viscosity of the haematocrit) is 0.45, *μ*_*Newtonian*_ is considered to be 4.06 × 10^−3^ (Pa·s).

### Doppler ultrasound measurement

We defined the uniform flow velocity in a circumferential direction at the inlet to be the mean of the blood flow velocity for the cardiac cycle obtained by averaging four consecutive cardiac cycles. Blood flow measurement as a preoperative examination for intra-arterial chemotherapy was conducted using a commercial ultrasound scanner (HDI 5000; ATL-Philips Medical Systems, WA). Doppler velocity measurement accuracy is ±1 % or 1/2 pixel. Doppler velocity measurements are most accurate when the acoustic beam is aligned parallel with blood flow. All the data for this study were obtained by an experienced sonographer.

In each patient, B-mode ultrasound and colour Doppler imaging showed that each artery had no severe stenosis, obstructive lesions or meandering. Using colour Doppler imaging, velocities were determined at the centrelines of the CCA and STA. Pulsatile waveforms were obtained at the centreline of the distal CCA approximately 1–2 cm before the bifurcation without relevant flow disturbances, and at the centreline of the STA, velocities were determined at the front of the ear. The sample volume was set to be 2/3 of the vessel lumen; the centre of the measurement site was in the long axis view; the angle correction was activated, and an angle of insonation of 60° was maintained whenever possible.

## Results

The simulations were conducted for 4 different types of vessel models (CCA, ICA, ECA and ECA branches). Each simulation took approximately 26 h (93,600 s) to complete.

**Fig. 6:**
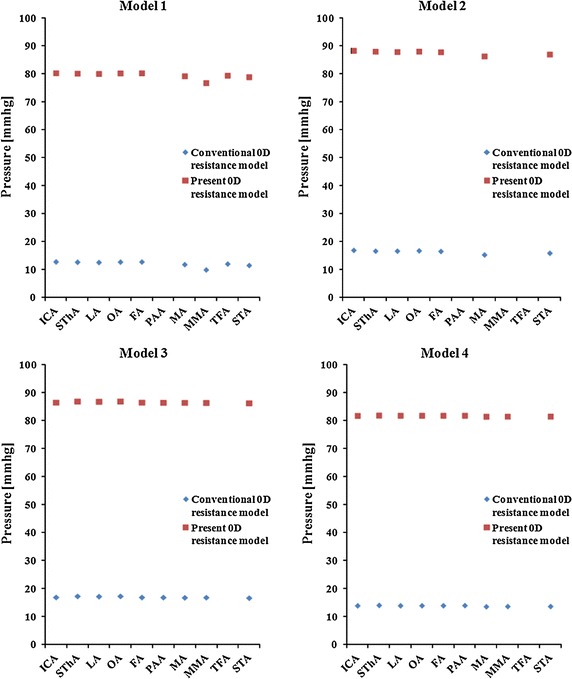
Pressure at the outlet of each blood vessel in the conventional 0D resistance model and in the present 0D resistance model. The pressure in the case of the present 0D resistance model was within the physiological range

**Fig. 7:**
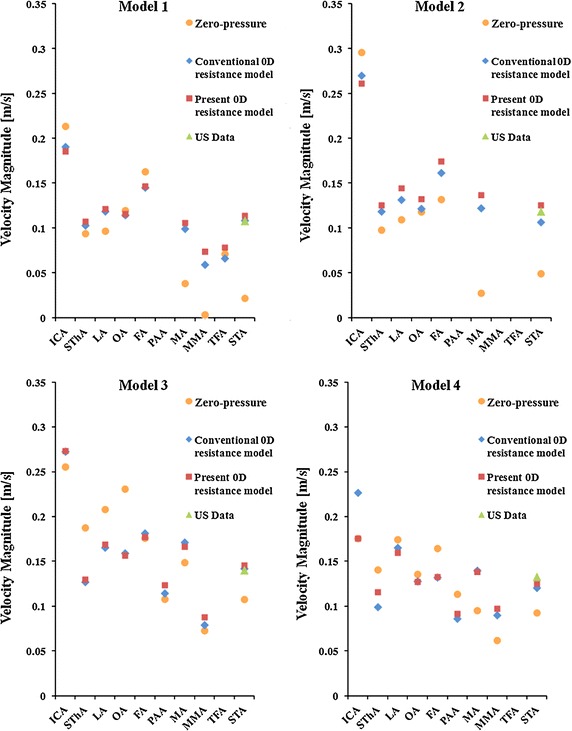
Velocity magnitude at the outlet of each blood vessel obtained with the zero-pressure outflow model, the conventional 0D model and the present 0D model. From the ultrasound data, only the velocity magnitude for the STA is shown

Figure 6 shows the results for the pressure at each outlet in the 0D simulation. The pressures obtained with the present 0D resistance model were within the physiological range. The velocity magnitudes at the outlet of each vessel are summarized in Fig. 7. The velocity magnitude for the STA as given by both 0D resistance models is in agreement with the ultrasound data.

**Fig. 8:**
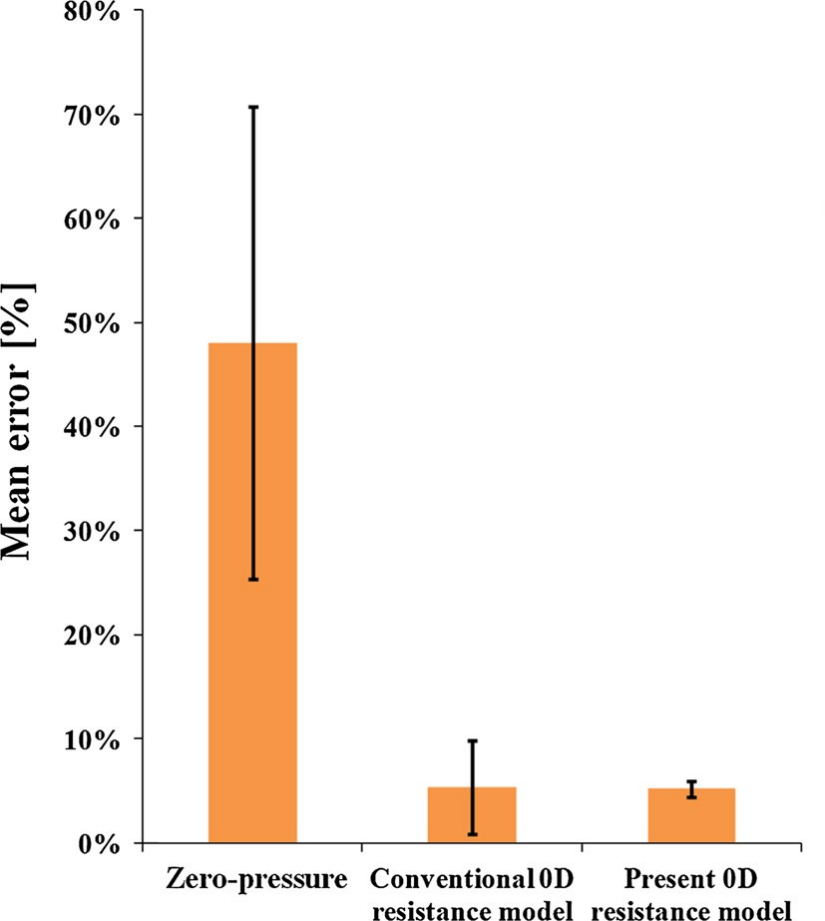
Mean error of the velocity for the STA obtained with the zero-pressure outflow model, the conventional 0D model and the present 0D model

To verify the simulation results, the blood velocities in the STA in each simulation were compared with those obtained from ultrasound measurements, as shown in Table 2. The mean error was defined as the average of the errors of the four models compared with the actual value obtained from ultrasound measurements, and the error value was calculated using the above-mentioned three types of boundary conditions. The mean error in the STA velocities in the simulation was 48.02 ± 22.66 %, 5.32 ± 4.52 % and 5.21 ± 0.78 % for the zero-pressure outflow model, the conventional 0D resistance model and the present 0D resistance model, respectively (Fig. 8). The simulation based on the zero-pressure outflow boundary condition yielded the largest mean error of the three models, whereas both 0D resistance models provided small mean errors. However, the standard deviation of the present 0D resistance model was smaller than that of the conventional 0D resistance model, which implies that simulation based on the present model is more precise.

**Table 2:** Velocities for the superficial temporal artery obtained for the four models with each outlet boundary condition together with ultrasound measurement data

	Model 1 (cm/s)	Model 2 (cm/s)	Model 3 (cm/s)	Model 4 (cm/s)
Zero-pressure outflow	2.167	4.917	10.760	9.260
Conventional 0D resistance model	10.834	10.643	14.196	12.049
Present 0D resistance model	11.368	12.538	14.565	12.525
Ultrasound data	10.796	11.854	14.016	13.304

## Discussion

This study focused on head and neck haemodynamics for the treatment of oral cancer, especially in cases where intra-arterial chemotherapy is necessary. There have been few patient-specific blood flow simulations for oral cancer chemotherapy to date. Although Rhode et al. [56] previously reported the results of simulations of haemodynamic flow in head and neck cancer chemotherapy, where a patient-specific vessel model was created from CT images, branches of the ECA, such as the OA, MA and STA, were not modelled, and the peripheral vascular network was not considered. Our present 0D resistance model is clinically useful because it can provide an accurate estimation of the pressure and the flow distribution in vessels. The application of the present 0D resistance model as a boundary condition yields more realistic blood flow simulations the results of which can be used to achieve optimal tumour control with minimum accompanying side effects in oral cancer chemotherapy. In this paper, we consider a novel method for haemodynamic flow simulation applicable to intra-arterial chemotherapy for oral cancer.

The usefulness of our peripheral blood vessel model (0D resistance model) was verified using a patient-specific 3D model of the carotid arteries and branches together with ultrasound data of the CCA (the inlet of the blood flow) and the ECA (the outlet of the blood flow). The zero-pressure outflow boundary condition underestimated the STA velocity (compared with the results of ultrasound measurements) with a mean error of 48.02 ± 22.66 %. Since the zero-pressure boundary condition considers none of the physiological effects of the peripheral vascular system, the flow distribution is determined by only the geometry of the analysis model constructed from medical images. On the other hand, the conventional and proposed 0D resistance models consider the effects of the circulatory system. Thus, the mean error of the STA velocities was drastically reduced to 5.32 ± 4.52 % and 5.21 ± 0.78 %, respectively. The results obtained with both 0D resistance models were in closer agreement with the measurement data than the results obtained using the free outflow boundary condition. Although the peripheral resistance in the present 0D resistance model tended to be greater than that in the conventional 0D resistance model, there was no significant difference in flow rate between the two 0D models. However, the present model correlated well with the experiment, yielding a standard deviation of 0.78 % compared with 4.52 % in the case of the conventional model. This improved precision may be because the present 0D resistance model determines *λ* automatically and systematically based on anatomical knowledge, while the conventional 0D model uses a fixed value (*λ* = 30).

This study assumes a healthy blood vessel, but angiogenesis has been observed in the region of tumour progression. Therefore, it may be not reasonable to assume the parameters are the same for healthy blood vessels in the region with the tumour and further studies should be performed in the future.

## Conclusions

We have presented some simple yet accurate outflow boundary conditions for conducting patient-specific blood simulations within a reasonable computation time for clinical applications. Since the region of interest in this study was the head and neck, the peripheral blood vessel network consists of mostly small blood vessels, which tend to be rigid. The outflow boundary conditions were designed based on the characteristics of the peripheral network by using the resistance of the blood vessel rather than the impedance, which combines compliance and resistance. To obtain an even more realistic simulation with the 0D model, the parameter *λ* was adjusted in accordance with anatomical knowledge.

The present 0D resistance model was used together with patient-specific 3D models of the carotid arteries to verify the simulation results, where ultrasound measurement results for the CCA and STA were used as reference data. The simulation based on the proposed 0D resistance model boundary conditions correlated well with the ultrasound measurement data and was more precise than those based on either the zero-pressure outflow condition or the conventional 0D resistance model. Furthermore, the pressure in the present 0D model was within the physiological range, in contrast to that of the conventional 0D model.

Our 0D resistance model is clinically useful because it can provide more realistic and accurate blood flow simulations, which may be helpful for achieving optimal tumour control with minimum accompanying side effects in oral cancer chemotherapy.

## Abbreviations

0D: zero dimension
ECA: external carotid artery
ICA: internal carotid artery
1D–0D: one dimensional–zero dimensional
3D: three-dimensional
STA: superficial temporal artery
CT: computed tomography
CCA: common carotid artery
DICOM: digital imaging and communication in medicine
SThA: superior thyroid artery
LA: lingual artery
FA: facial artery
OA: occipital artery
MA: maxillary artery
PAA: posterior auricular artery
MMA: middle meningeal artery
TFA: transverse facial artery
FVM: finite volume method
MR: magnetic resonance
COE: Centers of Excellence
